# Evaluation of Hardness and Retrogradation of Cooked Rice Based on Its Pasting Properties Using a Novel RVA Testing

**DOI:** 10.3390/foods10050987

**Published:** 2021-04-30

**Authors:** Sumiko Nakamura, Junji Katsura, Yasuhiro Maruyama, Ken’ichi Ohtsubo

**Affiliations:** 1 Faculty of Applied Life Sciences, Niigata University of Pharmacy and Applied Life Sciences, 265-1, Higashijima, Akiha-ku, Niigata 956-8603, Japan; snaka@nupals.ac.jp; 2 NSP Ltd., Nakanoki 2-31-5-B, Funabashi-shi, Chiba 274-0826, Japan; jkatsura@nsp-jpn.com (J.K.); yasuhiro-maruyama@nsp-jpn.com (Y.M.)

**Keywords:** amylopectin, rice quality, rapid visco analyzer, iodine absorption spectrum, starch

## Abstract

With rice being one of the most important crops worldwide, rapid and objective quality evaluation methods based on physicochemical measurements of rice are necessary. We compared the pasting properties of various rice samples using three different heating and cooling programs (maximum temperatures were 93, 120, and 140 °C, respectively) in a newly developed high-temperature-type Rapid Visco Analyzer (RVA, RVA 4800). Furthermore, we investigated the relationship between the different pasting properties measured by the three programs, with starch microstructure measured by iodine scanning analysis, the physical properties of the cooked rice measured by a Tensipresser after 2 h at 25 °C or after 24 h at 6 °C, and prolamin ratio measured by sodium dodecyl sulfate polyacrylamide gel electrophoresis (SDS-PAGE). The consistency value (final viscosity–minimum viscosity) yielded by a new program of maintenance for 2 min at 120 °C using RVA 4800 had a higher positive correlation with retrograded surface hardness H1(R) (*r* = 0.92), retrograded overall hardness H2(R) (*r* = 0.90), and the absorbance at λmax (Aλmax) of cooked rice (*r* = 0.88) and resistant starch (*r* = 0.80) than those by the conventional program at 93 °C. We developed estimation formulae for H1(R) for various kinds of rice, of which the determination coefficient was 0.86. It led to an easy and rapid assay method for the cooking properties of the various rice samples.

## 1. Introduction

Rice is one of the most important crops in the world, together with wheat and corn. Rice is used as table rice and for various food processing applications, such as sake (rice wine), rice snacks, and rice cake. The many rice cultivars grown in the world vary greatly in their cooking, sensory, and processing quality [1]. Rice-based foods have also been diversified, and convenient products, such as frozen cooked rice, extruded pregerminated brown rice, retort-pouch cooked rice, and aseptic cooked rice, have become increasingly available [2,3]. It, therefore, seems important and meaningful to develop a novel and simple method to evaluate the quality of rice products. As described by Juliano, rice quality evaluation includes eating quality assays for sensory evaluation and aroma testing as well as physical property measurements using, e.g., Instron, Texturometer, or Tensipresser, which are highlighted as indirect methods, in addition to amylose content, starch gelatinization temperature, gel consistency, amylography, and protein content determination [4]. Bhattacharya et al. classified the world’s 177 rice germplasms into 8 types (type I to type VIII) based on amylose, gel consistency, and Brabender relative breakdown [1]. Ohtsubo et al. reported on the quality assay of rice using traditional and novel tools, such as the measurements of amylose, physical properties, and pasting properties [5]. Bergman et al. reviewed rice end-use quality analysis, such as Apparent amylose content (AAC) and RVA analysis [1]. As starch properties, physical properties, and pasting properties of rice are closely related and affect each other, it is necessary to survey their correlations and apply multivariate analysis for evaluating palatability or processing suitability [1,6].

Starch comprises the most abundant component in rice grain and consists of amylose (linear α-1,4-glucan) and amylopectin (highly branched molecule with α-1,4 and α-1,6 bonds). AAC is measured by the iodine colorimetric method [7], and high-AAC rice becomes harder and nonsticky upon cooking [1]. AAC in waxy rice is lower than 2%, whereas common rice has AACs ranging from very low (5–12%), low (12–20%), and intermediate (20–25%) to high (25–33%) [8]. Bao reviewed that the starch structure is complex but can be divided into multiple levels, grouped from Level 1 to Level 6 [8]. Hizukuri et al. [9], Takeda et al. [10], and Hizukuri et al. [11] proposed that rice quality not only depends on amylose but also on amylopectin. Lian et al. investigated the identity of the main retrogradation-related properties of rice starch and reported that retrogradation rates of different rice starch showed a significant positive correlation with the proportion of the chains (degree of polymerization (DP) > 10) in amylopectin [12]. Recently, super-hard rice was developed by chemical mutation in Japan [13,14], the AAC of which is higher than 35% due to a high amount of super-long chains (SLCs) in amylopectin. Kubo et al. reported that *ae*/waxy double-mutant rice showed a higher pasting temperature and higher levels of resistant starch, which reveal that both AAC and amylopectin chain length affect the physical properties of rice [15]. Li et al. found that autoclave cooking, which is used to produce sticky “convenience rice”, affects sensory properties and increases leached amylopectin [16]. The chain length distributions of the debranched rice starches can be characterized by size-exclusion chromatography (SEC) or field flow fractionation (FFF) [8]. 

As Juliano [4] pointed out, the physical properties of cooked rice grains are important determinants of the eating quality of rice. Okadome et al. measured the physical properties by “low-compression high-compression with single cooked rice grains” using a Tensipresser [17,18]. Recently, a texture analyzer has been used for measuring the physical properties of cooked rice [19] or parboiled rice [20].

These days, in terms of the physical properties of cooked rice grains, not only the texture just after cooking but also the degree of retrogradation has become important because consumers often eat cooked rice after several hours or on the next day after cooking. Convenience stores and catering services use large-scale continuous rice cooking systems and provide cooked rice as a lunch box or take-out product. Therefore, we adopted a retrogradation test to measure the texture of Japanese and Chinese *Japonica* rice cultivars [21]. Traditional Japanese baked rice crackers include senbei and arare. Arare is a cracker made from rice cake, while senbei is a cracker-like snack made from cooked nonwaxy rice flour [22]. The degree of starch gelatinization rate and starch retrogradation are important factors for the quality of rice crackers [23].

The pasting properties of rice are useful indicators in the quality assay of cooked rice, rice cake, rice bread, and rice extrudate, for example. In several countries, such as Australia, China, Japan, and the United States, the RVA has become the standard method with which the rice processing industry and breeding programs determine rice pasting properties [1]. The RVA can provide various types of information, for example, indicators in cereal-based products, the apparent viscosity of cake batters and the quality of flour for cake making, interactions between starch and other compounds, extruded products and the measurement of the degree of cook, quality indicators for hydrocolloids and fibers, simulation and monitoring of processes, and enzymatic reactions [24]. Although the Brabender viscoamylograph has been used to assess rice pasting properties [1], Blakeney et al. [25] and Champagne et al. [26] showed that the RVA is useful in determining the “degree of cook” after the processing of rice into precooked and extruded products. Zhu et al. investigated the effect of soaking and cooking on the structure formation of cooked rice through thermal properties, dynamic viscoelasticity, and enzyme activity, in which they used the RVA for the measurements of enzyme activity [27]. We developed a novel estimation formula for AAC and resistant starch (RS) based on the pasting properties measured by an RVA [28], enabling the evaluation of the starch properties and processing suitability of material rice flours. Furthermore, we developed novel estimation formulae for oleic and linoleic acid contents based on the pasting properties of brown rice flours using an RVA, making it possible to predict easily and rapidly the nutritive and biofunctional characteristics of material rice [29].

To evaluate pasting properties, the behavior of rice starch at temperatures higher than 100 °C should be examined to elucidate the changes in rice quality after cooking and processing, such as extrusion cooking [30] or superheated moisture treatment [31]. In the present paper, we compare the pasting properties of various rice samples using three different types of heating and cooling programs on a newly developed high-temperature-type RVA (RVA 4800). We tried to differentiate the gelatinization and retrogradation properties of various rice starches more clearly by measuring the pasting properties at higher temperatures than the ordinary temperature used for a conventional RVA, 93 °C. Furthermore, we investigated the relationship between pasting properties and starch molecular structure, physical properties of cooked rice grains, and the prolamin ratio. Making use of RVA analysis, we were challenged to estimate the physical properties and retrogradation degree of the rice samples.

## 2. Materials and Methods

### 2.1. Materials

Glutinous rice varieties (red glutinous rice, Benika; purple glutinous rice, Shihou) were cultivated at the Niigata Prefectural Agricultural Research Institute in 2019. White glutinous rice varieties (Hakuchomochi, Koganemochi, Himenomochi, Kinunohada, and Kitayukimochi) were purchased in a local market.

Low-amylose *japonica* rice (Yumepirika), premium *japonica* rice (Koshihikari), medium-grain *japonica* rice, (Calrose), low-amylose *indica* rice (Jasmin rice), tropical *japonica* rice (Carnaroli), *japonica*-*indica* hybrid rice (Hoshiyutaka), and *indica* rice (Basmati) were purchased in a local market or online.

*Ae* mutant rice (Goami 2 and Dodam) were cultivated by Gyeonggi-do Agricultural Research and Extension Services in 2019 in Korea. Niigata 129gou was cultivated at the Niigata Prefectural Agricultural Research Institute in 2019.

### 2.2. Preparation of Polished White Rice Samples

Brown rice was polished using an experimental friction-type rice milling machine (Yamamotoseisakusyo, Co. Ltd., Tendoh, Japan) to obtain a milling yield (yield after polishing) of 90–91%. White rice flour was prepared using a cyclone mill (SFC-S1; Udy, Fort Collins, CO, USA) with a screen of 1 mm diameter pores.

### 2.3. Preparation of Starch Granules

Starch granules were prepared from polished rice flour using the cold alkaline method [32]. 

### 2.4. Iodine Absorption Spectrum

The iodine absorption spectrum of alkali-treated rice starch and cooked rice flour was measured using a UV-1800 spectrophotometer (Shimadzu Co. Ltd., Kyoto, Japan). The cooked rice samples were stored in a freezer at −80 °C. Subsequently, each sample was lyophilized using a freeze dryer (FD-1, Eyela, Tokyo Rikakikai Co., Ltd., Tokyo, Japan). The AACs of alkali-treated rice starch and cooked rice flours were estimated using Juliano’s iodine colorimetric method [7]. The iodine absorption spectrum was analyzed from 200 to 900 nm using a square cell with a 1 × 1 cm inner dimension [14]. The absorbance was measured at 620 nm (following Juliano’s method); we also measured peak wavelength on iodine staining (λ_max_), the molecular size of amylose and super-long chains (SLCs) of amylopectin, and absorbance at λ_max_ (Aλ_max_).

### 2.5. Pasting Properties

The pasting properties of milled rice flours from 17 rice cultivars were measured using an RVA (model Super 4; Newport Scientific Pty Ltd., Warriewood, Australia). Each sample (3.5 g based on 14% moisture content) was suspended in 25 mL of water. The measurement conditions were as follows: 1 min of heating at 50 °C, 4 min of heating from 50 to 93 °C, maintenance for 7 min at 93 °C, 4 min of cooling from 93 to 50 °C, and 3 min at 50 °C. The programmed heating and cooling cycle followed that of Toyoshima et al. [33].

The second program conditions were as follows: 1 min of heating at 50 °C, 6.5 min of heating from 50 to 120 °C, maintenance for 2 min at 120 °C, 6.5 min of cooling from 120 to 50 °C, and 3 min at 50 °C.

The third program conditions were as follows: 1 min of heating at 50 °C, 8.3 min of heating from 50 to 140 °C, maintenance for 3.3 min at 140 °C, 8.3 min of cooling from 140 to 50 °C, and 3 min at 50 °C.

### 2.6. Measurement of RS

The RS of cooked rice flour was measured according to the Association of Official Analytical Collaboration International (AOAC) method (2002.02), with a slight modification, using an RS assay kit (Megazyme, Ltd., Wicklow, Ireland). Each sample (100 mg; dry matter) was digested with pancreatin and amyloglucosidase at 37 °C for 6 h, and the glucose content was measured using a spectrophotometer at 510 nm.

### 2.7. Physical Properties of Cooked Rice Grains

For standard samples, milled rice (10 g) was added with 14 g of distilled water (1.4 times, *w/w*; standard moisture content, 13.5%; coefficient (gross water volume/dry matter weight): 1.77, calculated for each sample) in an aluminum cup. After soaking for 1 h, the samples were cooked in an electric rice cooker (National SR-SW182). The cooked rice samples were kept in the vessel at 25 °C for 2 h and then used to obtain measurements. The hardness and stickiness of the cooked rice grains were measured using a Tensipresser (My Boy System, Taketomo Electric Co., Tokyo, Japan) with the individual grain method in low compression (25%) and high compression (90%) tests [18]. The average of each parameter was calculated by measuring 20 individual grains.

As a staling test for cooked rice, the cooked samples were stored at 6 °C for 24 h and measured again with a Tensipresser according to the previously described method in low-compression (25%) and high-compression (90%) tests [21].

We defined retrograded hardness as the hardness of rice after storage at 6 °C for 24 h and the retrogradation degree as the hardness of rice after storage at 6 °C for 24 h divided by the hardness of rice after storage at 25 °C for 2 h.

We considered the hardness of the surface layer (=H1) and hardness of the overall layer (=H2), the stickiness of the surface layer (=S1), the stickiness of the overall layer (=S2), and the adhesion of the surface layer (=L3). Balance H1 (=S1/H1) means the ratio of stickiness to hardness of the surface layer, Balance H2 (=S2/H2) means the ratio of stickiness to hardness of the overall layer, Balance A1 (=A3/A1) means the ratio of adhesive work to hardness work of the surface layer, and Balance A2 (=A6/A4) means the ratio of adhesive work to hardness work of the overall layer.

### 2.8. Sodium Dodecyl Sulfate-Polyacrylamide Gel Electrophoresis (SDS-PAGE)

Protein was extracted from milled rice flour samples (0.5 g) by shaking with 2 mL of buffer A (50 mM Tris-HCl, pH 6.8, 2% SDS, 5% 2-mercaptoethanol) at 37 °C for 30 min and then centrifuging for 5 min at 3000× *g*. The supernatant (1 mL) was diluted with an equal volume of sample buffer (0.125 M Tris-HCl pH6.8, 10% 2-mercaptoethanol, 4% SDS, 10% sucrose, 0.004% bromophenol blue) and mixed well, followed by heating for 2 min at 100 °C. In total, 10 μg of extracted protein was loaded into each lane. SDS-PAGE was conducted with a 12% polyacrylamide gel, according to our previous report [21].

The values were calculated based on the intensities of various spots on the gel after SDS-PAGE analysis using the densitograph software library (CS Analyzer ver 3.0, ATTO Co., Tokyo, Japan).

### 2.9. Statistical Analyses

All results, including the significance of regression coefficients, were statistically analyzed using Student’s *t*-test, one-way analysis of variance, and Tukey’s test using Excel Statistics (ver. 2006; Microsoft Corp., Tokyo, Japan). Statistical analysis using an exponential function for RS was performed by XLSTAT 2021 for Microsoft Excel 2016.

## 3. Results and Discussion

### 3.1. Iodine Absorption Spectrum

Amylose is one component of rice starch that greatly affects the quality and gelatinization properties of cooked rice [1,8,9,10,34,35,36]. The amylose content of rice is controlled mainly by the *wx* gene (encoding granule-bound starch synthase I, *GBSS* *I*) [37,38,39]. Another starch component, amylopectin, is synthesized through the concerted reactions of starch synthase, starch branching enzyme, and debranching enzyme [40,41]. Umemoto et al. [42] showed that varietal differences in amylopectin chain-length distribution are regulated by functional variations in starch synthase (*SSIIa*). AAC comprises a large amount of amylose and a small amount of super long chain (SLC) amylopectin. Previous reports have described the molecular structures of several starches, including amylose and amylopectin branch chain lengths [43]. Starches in rice cultivars grown under low temperatures have significantly higher amylose content and lower SLC amylopectin content than cultivars grown under high temperatures [44,45]. Taira et al. [46] reported that the lipid content and fatty acid composition of rice are affected by daily mean temperature during ripening.

Igarashi et al. [47] reported a positive correlation between absorbance at λ_max_ and AAC. Inouchi et al. [48] and Jideani et al. [49] showed that the SLC content of starch can be estimated based on λ_max_ and the blue value of purified amylopectin.

We investigated the starch molecular structures of 17 kinds of rice samples, including glutinous, low-amylose japonica, low-amylose *indica*, *indica-japonica* hybrid, high-amylose, and *ae* mutant rice samples, according to the iodine colorimetric scanning method reported in our previous report [14].

Table 1 shows higher AAC in the starch of *ae* mutant rice cultivars (29.3–43.7%; mean, 37.3%) than the *japonica*-*indica* hybrid (25.3%), *indica* (23.7%), *japonica* (15.9–23.4%; mean, 18.6%), *japonica* low-amylose (15.5%), *indica* low-amylose (11.9%), and glutinous (0.5–4.3%; mean, 2.2%) rice cultivars.

The λ_max_ of *japonica*-*indica* hybrid rice starch (596.0 nm) was higher than those of *indica* (589.0 nm), *ae* mutant (576.5–593.5 nm; mean, 586.7 nm), *japonica* (579.5–590.5 nm; mean, 583.5 nm), *japonica* low-amylose (574.5 nm), *indica* low-amylose (565.5 nm), and glutinous (523.0 nm) rice cultivars. Although λ_max_ highly correlates with AAC, *ae* mutant rice showed high AAC and not so high λ_max_.

**Figure 1:**
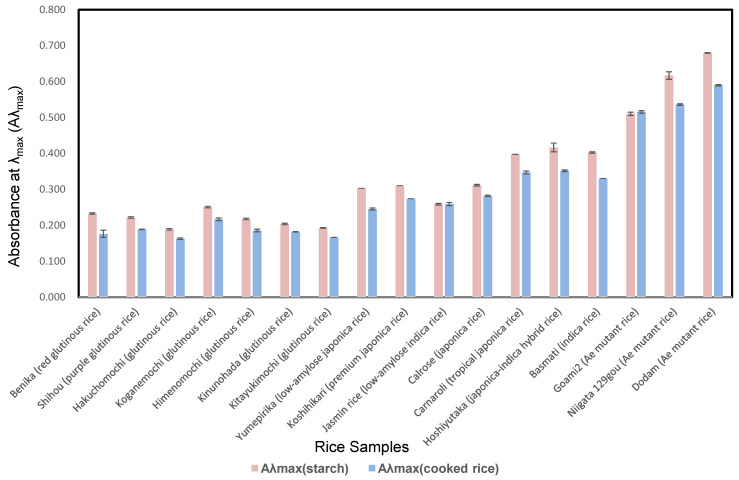
Aλ_max_ values of iodine absorption curve of starch and cooked rice of various kinds of cultivars.

The Aλ_max_ of the starch from *ae* mutant rice cultivars (0.510–0.680; mean, 0.602) was higher than those of *japonica*-*indica* hybrid (0.417), *indica* (0.403), *japonica* (0.310–0.397; mean, 0.340), *japonica* low-amylose (0.303), *indica* low-amylose (0.259), and glutinous (0.189–0.251; mean, 0.216) rice cultivars. Moreover, the Aλ_max_ of Koganemochi rice (0.251) was 1.3 times higher than that of Hakuchomochi rice (0.189), as shown in Figure 1. It is plausible that Kogenamochi retrograded more than Hakuchomochi because the Aλ_max_ of Koganemochi is higher than that of Hakuchomochi.

The Aλ_max_ values of *ae* mutant rice cultivars tend to be higher than those of other cultivars because the others contain SLC. It reveals that Aλ_max_ reflects not only the properties of amylose but also the effect of the amylopectin chain length; we selected Aλ_max_ as an indicator for the microstructure of starch [14].

In a previous study, we showed that AAC was negatively correlated with the λ_max_/Aλ_max_ ratio. The λ_max_/Aλ_max_ ratio of starch of glutinous rice (2087.9–2774.7; mean, 2452.2) was higher than those of *indica* low-amylose (2187.7), *japonica* low-amylose (1896.0), *japonica* (1487.4–1872.6; mean, 1740.1), *indica* (1463.4), *japonica*-*indica* hybrid (1431.5), and *ae* mutant (873.4–1130.4; mean, 987.0) rice cultivars.

In our previous study [14], we developed a formula for estimating amylopectin chain lengths with the degree of polymerization (DP ≥ 37), Fb_3_, on the basis of the iodine absorption curve of starch. In the present research, the Fb_3_ (DP ≥ 37) ratio of the starch of *ae* mutant rice cultivars (22.0–29.6%; mean, 25.8%) was higher than that of *japonica*-*indica* hybrid (17.8%), *indica* (17.2%), *japonica* (13.1%–17.0%; mean, 14.4%), *japonica* low-amylose (13.1%), *indica* low-amylose (10.8%), and glutinous (4.3–10.4%; mean, 7.4%) rice cultivars. Moreover, the Fb_3_ (DP ≥ 37) ratio of Koganemochi rice (10.4%) was 1.4 times higher than that of Hakuchomochi rice (7.7%).

Among the waxy rice, those suitable for producing soft cake, such as Hakuchomochi, have more short chains and fewer long chains than those used for hard cake, such as Koganemochi. Varietal differences are found in the amylopectin structure [50]. The hardening process in the manufacture of rice cakes is the most important process because it is time-consuming. For this reason, Koganemochi is considered the best waxy rice cultivar in Japan [51]. In the present study, we investigate the characterization of new glutinous rice cultivars and search for a novel way to maintain the hardness of starch even after cooking. In the present paper, we analyze the iodine absorption curve of both starch and cooked rice.

Table 1 shows that the absorbance values at 620 nm (representing AAC) of cooked *ae* mutant rice (0.466–0.551; mean, 0.498) were higher than those of other rice cultivars.

The λ_max_ of cooked *japonica*-*indica* hybrid rice was higher than those of other rice cultivars.

The Aλ_max_ values of the cooked *ae* mutant were higher than those of other rice cultivars. After cooking, the Aλ_max_ values tended to be lower when compared to starch, as shown in Figure 1.

The Fb_3_ (DP ≥ 37) ratios of cooked *ae* mutant rice were higher than those of other rice cultivars. Several studies have characterized the process of rice cake hardening [52,53,54]. The difference in amylopectin chain-length distribution causes differences in the rice cake hardening property [55]. The molar ratio of short chains to long chains of the amylopectin unit seemed to be one of the useful evaluation/selection indexes for the breeding of waxy rice [51].

In a comparison of iodine colorimetric parameters of starch and cooked rice, those of cooked rice tend to be less than those of starch [56]. This seems to be due to the decomposition of starch molecules during cooking. The degree of decomposition (lowering the ratio of the parameters of cooked rice to those of starch) is higher for glutinous and low-amylose rice than high-amylose rice and *ae* mutant rice.

In the present paper, we clarify the microstructures of rice starches using our novel and easy iodine method, the results of which will be useful to estimate processing suitability and the degree of retrogradation of various kinds of rice samples.

### 3.2. Pasting Properties

Pasting properties are a useful quality indicator because they affect the eating quality of rice [1,4,5]. The final viscosities (Fin. vis) of high-amylose rice cultivars have been shown to be higher than those of low-amylose cultivars, and Fin. vis is related to the degree of starch retrogradation during cooling [1,5]. A highly positive relationship was observed between SLC content and the consistency (Cons, Fin. vis–Mini. vis) of viscosity; therefore, SLCs in amylopectin appear to have a great effect on the Cons of starch [1]. When the temperature rises above the gelatinization temperature (GT) of the sample, starch granules begin to swell, and the viscosity increases. For waxy rice products, low-GT samples are preferred for desserts and rice cakes [57]. Takeda and Hizukuri [58] showed that amylose gelatinizes at higher temperatures than amylopectin. The resistance of amylose against gelatinization is considered an important factor in the gelatinization behavior of starch.

**Figure 2:**
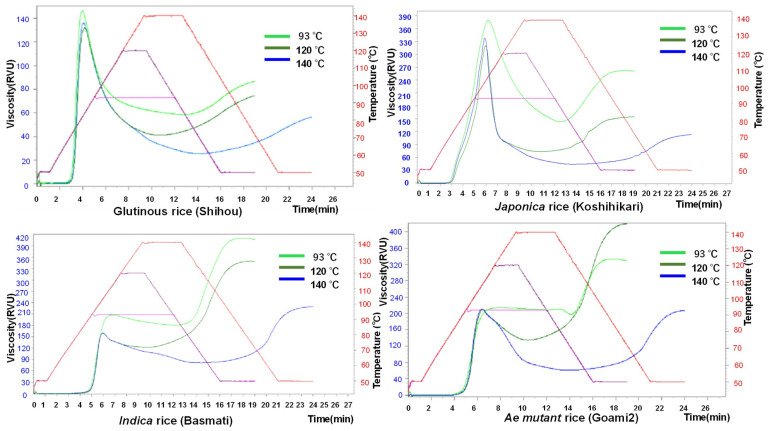
Pasting properties using three programs of RVA for various kinds of cultivars.

Here, we evaluate the relationship between the pasting properties by comparing three different programs: Program 1, maintenance for 7 min at 93 °C; Program 2, maintenance for 2 min at 120 °C; and Program 3, maintenance for 3.3 min at 140 °C using an RVA for 17 rice samples (Figure 2 and Table 2).

We used the same total time for the two programs (Program 1 and Program 2) because rapidity is indispensable for the RVA so as not to take more time even though a higher maximum temperature is set except for Program 3 (maintenance temperature is 140 °C).

As shown in Figure 2, the pasting properties of rice markedly change depending on the heating and cooling programs. Additionally, different groups of rice cultivars show various behavior for the different programs. The soft-type rice group, such as glutinous rice and *japonica* rice, showed higher maximum viscosities than final viscosities; on the contrary, the hard-type rice group, such as *indica* rice and *ae* mutant rice, showed higher final viscosities than maximum viscosities. Almost all rice groups showed the highest viscosities in Program 1 and the lowest viscosities in Program 3; intermediate viscosities were shown in Program 2. Nevertheless, only the *ae* mutant rice group showed the highest final viscosities in Program 2.

Table 2 shows that for Program 1, the Max. vis of *indica* low-amylose rice (485.2 RVU) was higher than those of *japonica* (379.0–411.9 RVU; mean, 396.3 RVU), *japonica* low-amylose (349.7 RVU), *japonica*-*indica* hybrid (233.3 RVU), *indica* (210.3 RVU), *ae* mutant (125.5–213.4 RVU; mean, 175.0 RVU), and glutinous (115.7–168.3 RVU; mean,134.2 RVU) rice cultivars; those for Program 2 and Program 3 showed similar trends. Furthermore, Max. vis tended to decrease as the maintenance temperature rose, as shown in Figure 2.

**Figure 3:**
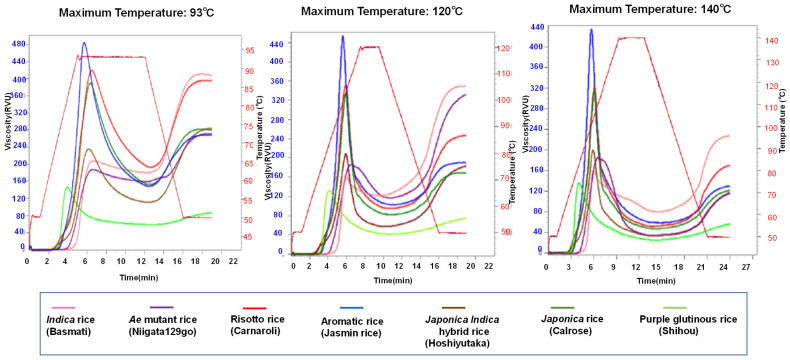
Pasting properties using three programs of RVA for various kinds of cultivars.

As shown in Figure 3, the second program (maintenance temperature 120 °C) is most suitable for differentiating the pasting properties of the various kinds of rice cultivars, from waxy to *ae* mutant rice. In the case of the conventional program (Program 1), the differentiation of final viscosity, the index for the degree of retrogradation, was not enough. Indica rice and risotto rice showed almost the same values, as did *japonica*, aromatic, and *ae* rice. Similarly, the final viscosities of aromatic, *japonica*, *japonica-indica*, and *ae* mutant rice showed almost the same values. We estimate that 93 °C is not high enough for hard-type rice to be completely gelatinized and 140 °C is a little too high, which leads to the inhibition of differentiation among the various rice starches.

The Fin. vis obtained with Program 1 for *indica* rice (411.8 RVU) was higher than those of other rice cultivars. Fin. vis in Programs 2 and 3 tended to decrease as the maintenance temperature rose in the samples, excluding *ae* mutant rice. The group of high-amylose starches includes two types of rice starches with similar apparent amylose content (AAC) but different super-long chain (SLC) content of amylopectin from *ae* mutant rice [59,60]. Fin. vis becomes higher by retrogradation of amylose. We estimated that the hydrogen bonds in the SLC of *ae* mutant rice were partially uncoupled at 120 °C, and the coupling was furthered by Program 3, which resulted in the lowest value. We estimated that the hydrogen bonds of the SLC of *ae* mutant rice were partially dissociated at 120 °C and almost completely dissociated at 140 °C.

The Cons result from Program 2 for *ae* mutant rice and glutinous rice was higher than those from Programs 1 and 3. Among the 17 rice samples, *ae* mutant rice showed the highest values and glutinous rice showed the lowest values, which agreed well with the phenomena that the cooked rice from *ae* rice retrogrades most and those from glutinous rice retrogrades least.

Among the glutinous rice samples, the Cons value and the Fin. vis. value of Koganemochi in Program 2 were markedly higher than those of Hakuchomochi, which agreed well with the retrogradation degree of waxy rice cake, as reported by Igarashi et al. [51].

The Cons values from the program at 120 °C for glutinous rice (21.6–52.3 RVU; mean, 32.5 RVU) were higher than those from the programs at 140 °C (21.3–52.5 RVU; mean, 30.7 RVU) and 93 °C (16.0–47.7 RVU; mean, 27.0 RVU). Glutinous rice showed the lowest value among the 17 rice samples. Furthermore, the Cons value of Koganemochi (52.3 RVU) in the program at 120 °C was 2.2 times higher than that of Hakuchomochi (23.8 RVU). Their Cons showed a similar trend with Fin. vis. It became clear that Cons is a useful parameter to differentiate the degree of retrogradation of various kinds of glutinous rice cultivars.

For the program at 93 °C, the Cons of *indica* rice (230.1 RVU) was higher than those of the programs at 120 °C (223.2 RVU) and 140 °C (147.3 RVU). Indica rice showed the second-highest value among the 17 samples.

**Figure 4:**
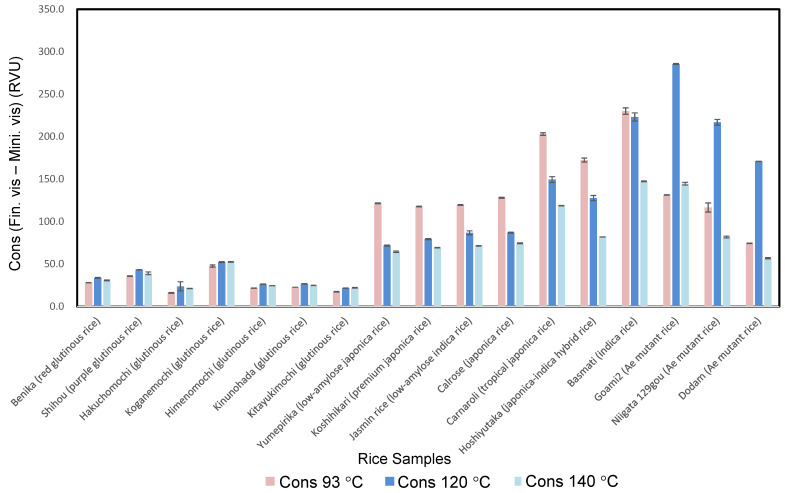
Consistency of pasting properties using three programs of RVA for various kinds of cultivars.

In this program, the Cons of *japonica*-*indica* hybrid rice (172.5 RVU) was higher than that of *japonica* (117.8–203.0 RVU; mean, 149.6 RVU), *japonica* low-amylose (121.5 RVU), and *indica* low-amylose (119.6 RVU) rice. Programs 2 and 3 showed a similar Cons trend, with the Mini. vis values decreasing as the maintenance temperature increases, as shown in Figure 4.

### 3.3. Physical Properties of Cooked Rice Grains

Table 3 shows the physical properties of cooked rice grains, evaluated by low-compression (25%) and high-compression (90%) methods [18] using a Tensipresser.

Table 3 shows that the hardness of the surface layer (H1) of *ae* mutant rice was higher than those of other rice cultivars. The hardness of the overall layer (H2) of *ae* mutant rice was higher than those of the other rice cultivars.

The stickiness of the surface layer (S1, sticky grains reveal high absolute values) of *japonica* rice was higher than those of the other rice cultivars, whereas the stickiness of the overall layer (S2) of *japonica* low-amylose rice was higher than those of the other rice cultivars.

The values for Balance H1, H2, A1, and A2 are important indices in evaluating the palatability of rice [18]. Balance A1 of glutinous rice was higher than those of the other rice cultivars, and the values of Balance H1, Balance H2, and Balance A2 showed a similar tendency.

*Japonica* low-amylose, *indica* low-amylose, *japonica,* and glutinous rice cultivars show soft and sticky eating qualities due to their physical properties of low hardness and high stickiness, while *indica*, *japonica*-*indica* hybrid, and *ae* mutant rice cultivars, with high amylose content, exhibit a texture of high hardness and low stickiness.

Low-amylose rice was found to be a stale-resistant rice cultivar, as many researchers have previously reported the staling characteristics of cooked low-amylose rice [61,62,63]. In the present investigation, the staling test for cooked rice was carried out by staling at 6 °C for 24 h. As shown in Table S1, the retrograded hardness of the surface layer H1(R) of *ae* mutant rice was higher than those of the other rice cultivars. Similarly, the retrograded hardness of the overall layer H2(R) of *ae* mutant rice was higher than those of the other rice cultivars. 

As a result of the staling test of cooked rice, *ae* mutant and *indica* rice showed very high hardness after staling, while glutinous, *japonica* low-amylose, and *indica* low-amylose rice showed low hardness and high stickiness (Table S1).

The H1(RD) of *indica* rice (1.90 times) was higher than that of *ae* mutant (1.48–1.57 times; mean, 1.52 times), *japonica* (1.34–1.58 times; mean, 1.42 times), *japonica*-*indica* hybrid (1.37 times), *japonica* low-amylose (1.26 times), *indica* low-amylose (1.20 times), and glutinous (0.78–1.21 times; mean, 0.98 times) rice cultivars. 

The H2(RD) of *ae* mutant rice was higher than those of the other rice cultivars; moreover, the H1(RD) of Koganemochi rice (1.21 times) was higher than that of Hakuchomochi rice (0.93 times). The retrogradation degree of hardness of *ae* mutant rice and *indica* rice showed very high values, while those of glutinous rice showed very low values.

As Umemoto et al. [64] showed, the variation in *SS IIa* (starch synthase IIa gene) affects eating quality after the storage of cooked rice at 5 °C; high-amylose and high-SLC rice cultivars seem to retrograde more markedly than low-amylose rice cultivars.

### 3.4. RS Content

Resistant starch (RS) is classified into four categories: (1) RS1, in whole-grain flour and unpolished rice, (2) RS2, a high-amylose content and high-crystallinity starch, (3) RS3, a retrograded starch produced by cooling gelatinized starch, and (4) RS4, chemically modified starch [65].

**Figure 5:**
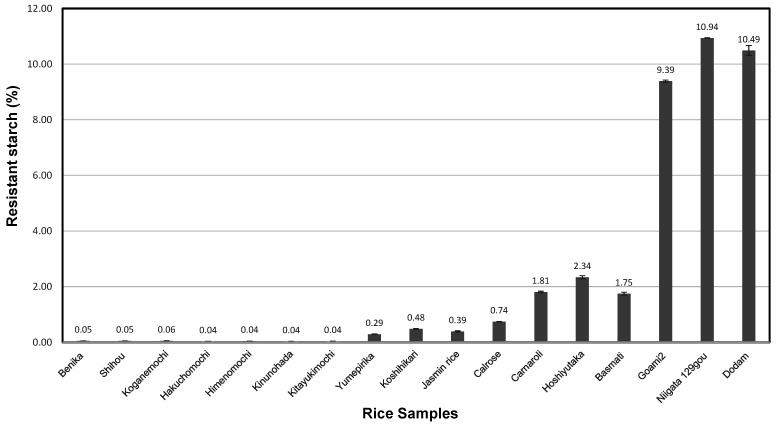
Resistant starch of various kinds of cooked rice after 24 h at 6 °C.

As shown in Figure 4 and Figure 5, the RS contents of *ae* mutant rice (9.39–10.94%; mean, 10.28%) were higher than those of *japonica*-*indica* hybrid (2.34%), *indica* rice (1.75%), *japonica* (0.48–1.81%; mean, 0.88%), *indica* low-amylose (0.39%), *japonica* low-amylose (0.29%), and glutinous (0.04–0.06%; mean, 0.05%) rice cultivars. *Ae* mutant rice grains had markedly higher amounts of RS than the other rice cultivars even after cooking. Yang et al. [66] reported that mutant rice is rich in RS. The *japonica* rice cultivars had significantly lower RS contents than the *indica* rice and *japonica-indica* hybrids with similar amylose content [14].

In general, starches rich in amylose are naturally more resistant to digestion and more susceptible to retrogradation, while the SLCs in amylopectin behave in a manner similar to amylose by restricting starch swelling [67,68]. The *japonica* rice cultivars had significantly lower RS contents than *indica* and *japonica-indica* hybrid rice cultivars with similar amylose content [14,69,70].

Two crystalline structures of starch (A and B types) that contain different proportions of amylopectin have been identified. A-type starches are found in cereals, whereas B-type starches are found in tubers and amylose-rich starches [71].

In general, amylopectin retrogradation significantly increases the amount of RS [12]. RS content seems to be important, as increased RS yields foods with greater biofunctional qualities, such as low-glycemic-index rice for people with diabetes [72,73,74,75,76,77].

### 3.5. SDS PAGE

Protein is the second-most abundant constituent of milled rice after starch. The physical properties of cooked rice grains are affected not only by starch but also by protein contents and their composition. Rice seed storage proteins consist mainly of glutelins and prolamins, such as 13 kDa prolamin [78,79]. The higher the protein content, the harder and less sticky the rice upon cooking [80].

The protein content in rice grains is influenced by weather conditions [81]. Protein production also tends to increase with higher levels of nitrogenous fertilizer at any planting density [82]. As shown in Figure S1, the intensities of the spots of 13 kDa prolamin reveal the prolamin ratios of Benika (20.2%), Shihou (18.9%), Hakuchomochi (19.6%), Koganemochi (19.6%), Himenomochi (21.4%), Kinunohada (22.3%), Kitayukimochi (22.0%), Yumepirika (23.8%), Koshihikari (23.4%), Jasmin (27.3%), Calrose (27.0%), Carnaroli (24.1%), Hoshiyutaka (24.0%), Basmati (26.6%), Goami 2 (23.7%), Niigata 129gou (23.2%), and Dodam (24.3%).

The glutinous rice cultivars (18.9–22.3%; mean, 20.5%) had lower 13 kDa prolamin ratios than *indica* low-amylose (27.3%), *indica* (26.6%), *japonica* (23.4–27.3%; mean, 24.5%), *japonica-indica* hybrid (23.9%), *ae* mutant (23.2–24.3%; mean, 23.7%), and *japonica* low-amylose (23.4%) rice cultivars.

### 3.6. Correlations between the Pasting Properties of Rice from the Three Programs with the Results of Cooked Rice Characterization

The pasting properties also influence the rice eating quality; therefore, it is useful to conduct tests of gelatinization properties in a quality assay for rice. AAC contains high levels of amylose and low levels of SLC in amylopectin. Generally, low-amylose rice becomes soft and sticky upon cooking, while high amylose rice becomes hard and separated [83].

H1(R) showed a positive correlation with Cons for the programs at 93 °C (*r* = 0.53; *p* < 0.05), 120 °C (*r* = 0.92; *p* < 0.01), and 140 °C (*r* = 0.67; *p* < 0.01). Furthermore, H2(R) showed a similar trend.

H2(RD) showed a positive correlation with Cons for the program at 93 °C (*r* = 0.47), 120 °C (*r* = 0.72; *p* < 0.01), and 140 °C (*r* = 0.56; *p* < 0.01). Furthermore, S2(RD) showed a negative correlation with Cons for the program at 93 °C (*r* = −0.57; *p* < 0.05), 120 °C (*r* = −0.92; *p* < 0.01), and 140 °C (*r* = 0.72; *p* < 0.01), as shown in Table 4. As shown in Table 3, the second program (120 °C) showed the highest correlation with the physical properties of cooked rice grains and starch microstructure. In a previous study, we reported that pasting properties are correlated to the texture of cooked rice grains [28]. These consistency values from Programs 2 and 3 using an RVA enable us to easily and rapidly evaluate the physical property of various kinds of cooked rice.

The Aλ_max_ of cooked rice showed a positive correlation with Cons for Program 1 (*r* = 0.50; *p* < 0.05), Program 2 (*r* = 0.88; *p* < 0.01), and Program 3 (*r* = 0.61; *p* < 0.01). Furthermore, the Aλ_max_ of starch showed a similar trend, as shown in Table 4. In our previous study, we reported that pasting properties are correlated to the AAC of various kinds of starch or milled rice [14,28]. AAC is higher than the actual amylose content because of the long-chain amylopectin binding with iodine. SLC in amylopectin appears to have a great effect on the consistency of starch, as reported in our previous study [14].

The RS of cooked rice showed a positive correlation with Cons for Program 2 (*r* = 0.80; *p* < 0.01), as shown in Table 4. Cons for Program 2 using an RVA enables us to evaluate the RS of various kinds of cooked rice by an easy and rapid RVA analysis. 

**Figure 6:**
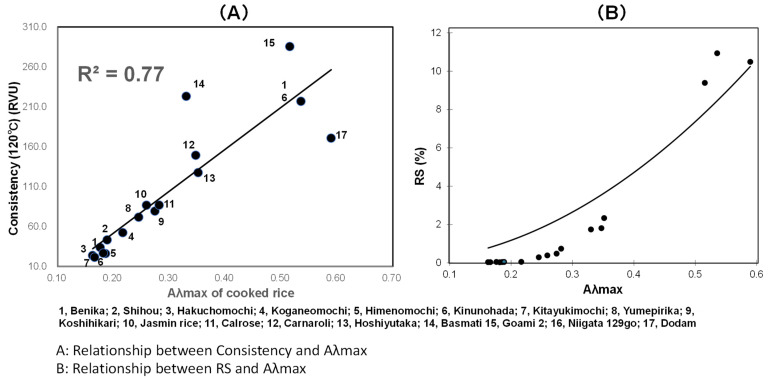
Relationship between starch microstructure (**A**) and pasting properties (**B**).

As shown in Figure 6A, Cons for Program 2 showed a high correlation with Aλmax of cooked rice by linear regression analysis; R^2^ was 0.77. Additionally, based upon the estimation of Aλmax, RS can be estimated by the analysis using an exponential function, as shown in Figure 6B; the estimation formula is revealed below.

RS = −9.37 × 10^6^ × 0.16/((−9.37 × 10^6^ − 0.16) × exp (−7.41 × Aλmax) + 0.16) (1)

According to the abovementioned equation, the mean squared error is 1.38 and the RMSE (root mean squared error) is 1.18.

It is possible to estimate RS using Aλmax as a parameter without using the labor-consuming enzyme method.

The 13 kDa prolamin ratio showed a positive correlation with Cons for Program 1 (*r* = 0.74; *p* < 0.01), Program 2 (*r* = 0.51; *p* < 0.05), and Program 3 (*r* = 0.62; *p* < 0.01). It is well known that rice with high protein content shows inferior palatability. Matsui et al. [78] showed that the final viscosity and consistency of near-isogenic line pairs for the *low glutelin content1* (*Lgc1*) gene locus were significantly higher in low-glutelin lines; moreover, surface stickiness was significantly decreased in the low-glutelin lines.

In the present investigation, the Cons values for a new Program 2 using RVA had a higher correlation with the retrogradation of hardness, stickiness, and RS values of various kinds of cooked rice than the conventional program at 93 °C.

We also investigated the correlation of pasting property with starch molecular structure and physical property using narrow-range *japonica* low-amylose rice cultivars for commercial use in Japan. Differently from the wide range of various rice samples, from waxy rice to *ae* mutant rice samples, Aλ_max_, an indicator for the microstructure of starch by iodine scanning analysis, did not show a significant correlation; however, λ_max_ showed a significant correlation with pasting property (Cons; *r* = 0.45, *p* < 0.05) and hardness of the cooked rice grains (*r* = 0.46, *p* < 0.05) in the case of narrow-range *japonica* rice cultivars for commercial use in Japan. The reason for this difference is that SLC differs markedly for the wide range of rice samples, from waxy rice to *ae*-mutant rice; on the contrary, narrow-range *japonica* low-amylose rice, such as Japanese rice for commercial use, differs in amylose, mainly in content (λ_max_), but does not differ in the SLC of amylopectin (Aλ_max_).

### 3.7. Formulae for Estimating the Retrograded Hardness H1(R) of Various Kinds of Cooked Rice Based on the Program at 120 °C Using an RVA

In our previous paper, we developed a novel estimation formula for the balance degree of the surface layer (A3/A1) based on the iodine absorption curve of milled rice [56]. 

**Figure 7:**
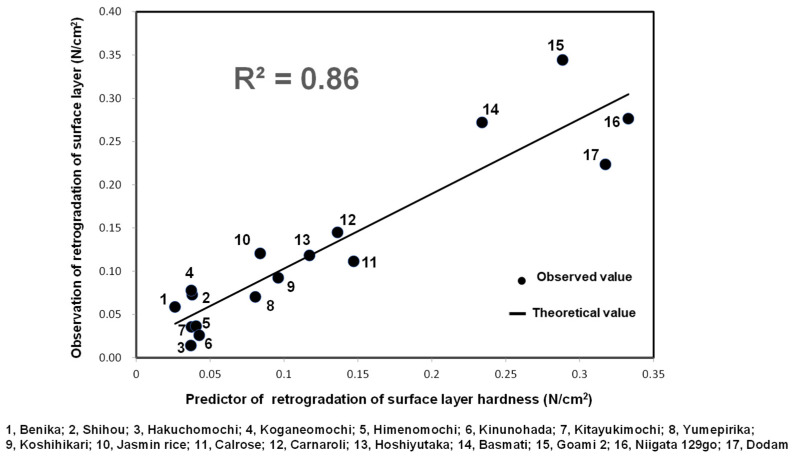
Formula for estimating the H1 (R) of various kinds of cooked rice based on Program 2 (120 °C).

The retrogradation of starch is responsible for the hardening of processed cooked rice, rice cakes, and crackers [84]. Figure 7 shows the formula developed to estimate H1(R) based on the pasting properties of various kinds of rice samples using the novel Program 2. The equation had a determination coefficient (R^2^) of 0.86 based on the calibration. The following formula for estimating H1(R) was obtained using 17 varieties of rice for calibration (1, Benika; 2, Shihou; 3, Hakuchomochi; 4, Koganemochi; 5, Himenomochi; 6, Kinunohada; 7, Kitayukimochi; 8, Yumepirika; 9, Koshihikari; 10, Jasmine rice; 11, Calrose; 12, Carnaroli; 13, Hoshiyutaka; 14, Basmati; 15, Goami 2; 16, Niigata 129gou; 17, Dodam):

H1(R) (N/cm^2^) = 90.42 × Cons + 433.2 × Pt (2)

where Pt is the pasting temperature. This formula will enable us to rapidly and easily estimate the H1(R) of various kinds of cooked rice based on the novel Program 2 using an RVA.

### 3.8. Formulae for Estimating H1(RD) of Cooked Glutinous Rice Based on the Program at 120 °C Using an RVA

In measuring the pasting property of glutinous rice with an RVA, copper sulfate is usually used to prevent the effects of endogenous amylase activity. Copper sulfate is a deleterious substance; therefore, Sugiura et al. [85] used an RVA for measuring rice cake hardness with a 3% sodium chloride solution. Several studies related to the assessment of rice cake hardening have been reported [50,52,53]. 

The hardness of glutinous rice is important in order to improve working efficiency in the manufacture of rice cakes and crackers. Okamoto and Nemoto [53] tried to establish a method to estimate rice cake hardness of upland rice using an RVA with a 400-ppm solution of copper sulfate. Gelatinization temperature is related to the hardening speed of rice cake, and glutinous rice cultivars with higher gelatinization temperatures are more suitable for rice cake processing [54,84,86].

**Figure 8:**
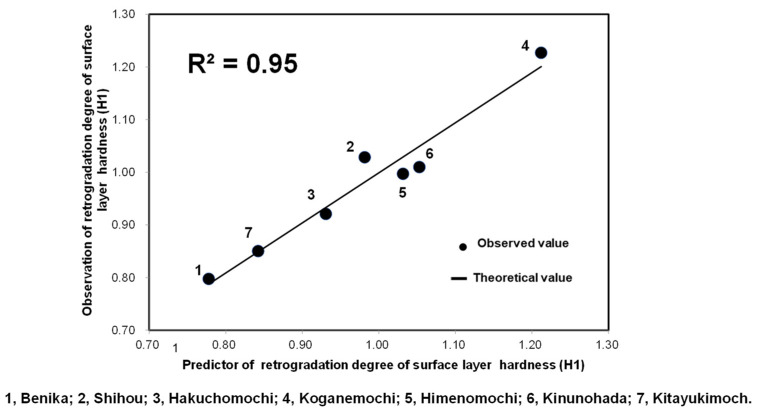
Formula for estimating H1 (RD) of cooked rice and glutinous rice based on Program 2 (120 °C).

Figure 8 shows the formula developed for estimating H1(RD) using the pasting properties of glutinous rice based on the novel program at 120 °C. The equation had a determination coefficient (R^2^) of 0.95 based on the calibration. The following formula for estimating H1(RD) was obtained using seven glutinous rice varieties for calibration (1, Benika; 2, Shihou; 3, Hakuchomochi; 4, Koganemochi; 5, Himenomochi; 6, Kinunohada; 7, Kitayukimochi):

H1(RD) = 0.012 × Mini. vis − 0.079 × Pt + 0.007 × SB − 6.155(3)

where SB represents a setback. This formula enables us to evaluate the H1(RD) of cooked rice or rice cake made from glutinous rice, based on the novel Program 2 using an RVA.

In the present investigation, we have proposed an easy and rapid method to estimate rice quality using iodine colorimetric analysis, texture measurements, and pasting properties by a new-type RVA.

We have reported the novel estimation formulae to estimate the fatty acid composition of brown rice using RVA analysis [29]. RVA and other physicochemical measurements will be useful to estimate the biofunctional properties of rice in the near future.

By the combination of physicochemical measurements, sensory tests, and genotyping, the ability to estimate or classify rice quality towards diversified consumer needs in the future is improved.

## 4. Conclusions

We have made improvements to analyzing pasting properties in a new program using RVA 4800, and novel estimation formulae were developed in this paper for estimates of the retrograded hardness of cooked rice H1(R) and the degree of retrogradation H1(RD), which has led to an easy and rapid evaluation of the cooking qualities of various rice samples. The following are the main features:

1. Among the three kinds of RVA programs, Program 2 (120 °C) showed the highest correlations with starch microstructure (Aλ_max_), RS, physical properties, and degree of retrogradation of the cooked rice grains.
2. The novel Program 2 (120 °C) showed high determination coefficients for hardness and the degree of retrogradation of cooked rice grains.
3. The novel RVA Program 2 enables us to easily and rapidly estimate the cooking and processing characteristics of various kinds of rice cultivars.

## Figures and Tables

**Table 1:** The iodaine sbsorption curve of starch and cooked rice of various kinds of rice.

	Starch																Cookedrice														
	AAC	Absorbance		SD	λ_max_		SD	Aλ_max_		SD	λ_max_/Aλ_max_		SD	Fb3(%)		SD	Absorbance		SD	λ_max_		SD	Aλ_max_		SD	λ_max_/Aλ_max_		SD	Fb3 (%)		SD
	(%)	620 nm															620 nm														
Benika (red glutinous rice)	3.5	0.122	b	0.1	523.0	a	0.0	0.233	b	0.002	2249.6	g	20.5	4.7	a	0.1	0.081	b	0.006	523.0	a	0.0	0.176	b	0.010	3016.1	g	169.6	4.0	a	0.4
Shihou (purple glutinous rice)	2.9	0.113	b	0.2	523.0	a	0.0	0.222	b	0.002	2361.3	g	22.6	4.3	a	0.1	0.084	b	0.000	523.0	a	0.0	0.189	b	0.001	2811.7	g	10.5	3.8	a	0.0
Hakuchomochi (glutinous rice)	0.5	0.083	a	0.1	523.0	a	0.0	0.189	a	0.002	2774.7	h	31.2	7.7	b	0.1	0.070	a	0.001	523.0	a	0.0	0.163	a	0.002	2767.2	g	31.3	6.5	b	0.1
Koganemochi (glutinous rice)	4.3	0.132	c	0.1	523.0	a	0.0	0.251	c	0.002	2087.9	f	17.7	10.4	d	0.1	0.112	c	0.002	523.0	a	0.0	0.217	c	0.004	2083.7	e	17.7	8.9	d	0.2
Himenomochi (glutinous rice)	2.2	0.104	b	0.1	523.0	a	0.0	0.218	b	0.002	2404.7	g	23.5	8.9	c	0.1	0.086	b	0.001	523.0	a	0.0	0.186	b	0.004	2399.1	f	23.4	7.5	c	0.2
Kinunohada (glutinous rice)	1.3	0.093	a	0.1	523.0	a	0.0	0.204	a	0.002	2570.2	g	26.8	8.3	c	0.1	0.082	b	0.001	523.0	a	0.0	0.182	b	0.001	2563.7	f	26.9	7.3	c	0.0
Kitayukimochi (glutinous rice)	0.7	0.085	a	0.1	523.0	a	0.0	0.193	a	0.001	2716.9	h	10.0	7.8	b	0.0	0.072	a	0.000	523.0	a	0.0	0.166	a	0.000	2709.8	g	9.9	6.6	b	0.0
Yumepirika (low-amylose *japonica* rice)	15.5	0.214	d	0.0	574.5	b	0.7	0.303	d	0.000	1896.0	e	2.3	13.1	e	0.0	0.203	d	0.003	555.5	b	2.1	0.245	d	0.003	2267.4	f	17.5	10.2	e	0.1
Koshihikari (premium *japonica* rice)	15.9	0.286	e	0.0	580.5	b	2.1	0.310	d	0.000	1872.6	e	6.8	13.1	e	0.0	0.227	e	0.001	558.0	b	2.8	0.274	e	0.000	2036.5	d	10.3	11.5	f	0.0
Jasmin rice (low-amylose *indica* rice)	11.9	0.278	e	0.3	565.5	c	2.1	0.259	c	0.002	2187.7	g	9.7	10.8	d	0.1	0.204	d	0.004	549.5	b	0.7	0.259	e	0.004	2121.9	e	37.5	10.8	e	0.2
Calrose (*japonica* rice)	16.5	0.299	e	0.2	579.5	b	3.5	0.312	d	0.002	1860.4	e	24.0	13.1	e	0.1	0.240	e	0.001	560.0	b	4.2	0.282	e	0.002	1989.3	d	0.1	11.8	f	0.1
Carnaroli (tropical *japonica* rice)	23.4	0.379	f	0.1	590.5	d	2.1	0.397	e	0.000	1487.4	d	5.3	17.0	f	0.0	0.328	f	0.006	582.0	d	2.8	0.347	f	0.004	1677.3	b	12.4	14.7	g	0.2
Hoshiyutaka (*japonica-indica* hybrid rice)	25.3	0.374	f	0.8	596.0	d	2.8	0.417	e	0.012	1431.5	d	34.5	17.8	f	0.5	0.329	f	0.002	582.5	d	2.1	0.352	f	0.002	1657.2	b	4.0	14.9	g	0.1
Basmati (*indica* rice)	23.7	0.375	f	0.2	589.0	d	2.8	0.403	e	0.002	1463.4	d	0.7	17.2	f	0.1	0.301	f	0.001	574.5	c	0.7	0.330	f	0.000	1740.9	c	2.1	14.0	g	0.0
Goami2 (*Ae* mutant rice)	29.3	0.471	g	0.4	576.5	b	0.7	0.510	f	0.004	1130.4	c	8.0	22.0	g	0.2	0.466	g	0.002	575.5	c	0.7	0.516	g	0.004	1116.4	a	9.0	22.3	h	0.2
Niigata 129gou (*Ae* mutant rice)	39.0	0.590	h	0.7	590.0	d	1.4	0.617	g	0.011	957.2	b	18.8	25.9	g	0.4	0.477	g	0.004	572.5	c	0.7	0.536	g	0.002	1069.1	a	5.6	23.2	h	0.1
Dodam (*Ae* mutant rice)	43.7	0.640	i	0.1	593.5	d	2.1	0.680	h	0.001	873.4	a	4.0	29.6	h	0.0	0.551	h	0.001	582.5	d	2.1	0.590	h	0.002	988.1	a	7.2	25.6	h	0.1

Within each measure (aborbance, λmax, etc.) in the same column, different letters (a, b, c, etc.) denote statistically significant differences.

**Table 2:** Pasting properties using three programs of RVA for various kinds of cultivars.

Samples	Max.vis.			Mini.vis.			BD			Final.vis			SB			Pt			Cons		
	(RVU)		SD	(RVU)		SD	(RVU)		SD	(RVU)		SD	(RVU)		SD	(°C)		SD	(RVU)		SD
Benika (red glutinous rice)	145.5	c	1.4	58.0	b	0.5	87.6	c	0.8	85.9	b	0.7	−59.6	d	0.6	50.6	a	0.1	28.0	b	0.2
Shihou (purple glutinous rice)	168.3	d	0.6	79.3	b	0.6	88.9	c	1.2	115.1	b	0.1	−53.1	d	0.6	50.5	a	0.1	35.8	c	0.5
Hakuchomochi (glutinous rice)	156.3	d	0.1	30.9	a	0.6	125.4	d	0.5	46.9	a	0.2	−109.4	b	0.0	50.2	a	0.1	16.0	a	0.5
Koganemochi (glutinous rice)	222.7	b	2.6	99.5	c	2.4	123.2	d	0.2	147.2	c	1.2	−75.5	c	0.7	53.6	a	4.3	47.7	c	1.2
Himenomochi (glutinous rice)	115.7	a	0.5	41.2	a	0.2	74.5	b	0.3	62.8	a	0.4	−53.0	d	0.1	50.7	a	0.1	21.6	b	0.1
Kinunohada (glutinous rice)	115.9	a	0.6	44.1	a	0.1	71.8	b	0.5	66.8	a	0.1	−49.1	d	0.3	50.7	a	0.0	22.7	b	0.0
Kitayukimochi (glutinous rice)	130.8	b	2.8	32.6	a	0.8	98.2	c	1.9	49.9	a	0.5	−80.9	c	1.1	50.5	a	0.1	17.3	a	0.4
Yumepirika (low-amylose *japonica* rice)	349.7	f	0.7	143.4	d	0.6	206.3	e	1.3	264.9	d	0.2	−84.8	c	0.9	66.8	c	0.5	121.5	e	0.4
Koshihikari (premium *japonica* rice)	379.0	f	0.6	142.4	d	0.8	236.7	e	0.1	260.1	d	1.2	−118.9	b	0.6	66.9	c	0.2	117.8	e	0.5
Jasmin rice (low-amylose *indica* rice)	485.2	g	1.9	147.6	d	1.6	337.6	f	3.6	267.2	d	1.2	−218.0	a	3.1	70.9	c	0.4	119.6	e	0.5
Calrose (*japonica* rice)	398.0	g	11.7	152.1	d	0.6	245.9	e	12.3	280.2	d	0.1	−117.7	b	11.7	68.9	c	0.0	128.1	e	0.6
Carnaroli (tropical *japonica* rice)	411.9	g	10.7	191.3	e	2.7	220.6	e	8.0	394.4	e	1.1	−17.5	e	9.6	65.5	c	0.1	203.0	g	1.6
Hoshiyutaka (*japonica-indica* hybrid rice)	233.3	e	3.1	106.5	c	5.3	126.8	d	2.2	279.0	d	7.6	45.7	f	4.5	61.5	b	1.6	172.5	f	2.3
Basmati (*indica* rice)	210.3	e	3.4	181.7	e	2.8	28.6	a	0.5	411.8	e	6.6	201.5	h	3.2	76.7	d	0.5	230.1	h	3.8
Goami2 (*Ae* mutant rice)	213.4	e	0.5	197.0	e	0.9	16.5	a	0.1	328.3	d	0.6	114.9	g	0.3	78.5	d	1.4	131.3	e	0.2
Niigata 129gou (*Ae* mutant rice)	186.1	e	1.8	158.3	d	0.5	27.7	a	1.3	274.9	d	4.8	88.8	g	6.6	79.7	d	0.3	116.6	e	5.3
Dodam (*Ae* mutant rice)	125.5	b	0.0	99.5	c	0.1	26.0	a	0.1	173.8	c	0.1	48.3	f	0.2	78.9	d	0.1	74.3	d	0.2
Benika (red glutinous rice)	134.7	b	3.8	40.9	b	0.2	93.8	c	4.0	74.7	b	0.4	−60.0	d	3.5	68.7	c	0.0	33.8	b	0.5
Shihou (purple glutinous rice)	158.1	c	0.9	61.4	c	0.5	96.7	c	0.5	104.8	b	0.5	−53.3	d	0.5	70.0	c	2.5	43.3	c	0.0
Hakuchomochi (glutinous rice)	144.1	b	2.5	21.4	a	1.0	122.8	d	3.5	45.2	a	4.4	−99.0	c	1.8	60.5	a	0.4	23.8	a	5.4
Koganemochi (glutinous rice)	206.2	d	0.1	85.0	d	0.7	121.3	d	0.6	137.3	c	0.2	−68.9	d	0.1	69.3	c	3.5	52.3	c	0.5
Himenomochi (glutinous rice)	106.8	a	1.2	29.0	a	0.1	77.8	b	1.1	55.2	a	0.3	−51.6	d	0.9	65.0	b	0.5	26.2	a	0.2
Kinunohada (glutinous rice)	107.0	a	1.5	30.3	a	0.7	76.7	b	0.9	57.0	a	0.9	−50.0	d	0.6	65.1	b	2.9	26.7	a	0.3
Kitayukimochi (glutinous rice)	121.0	a	2.8	22.8	a	0.5	98.2	c	2.2	44.4	a	0.4	−76.6	d	2.4	63.7	b	0.1	21.6	a	0.1
Yumepirika (low-amylose *japonica* rice)	278.5	e	1.2	63.6	c	0.3	214.9	e	1.5	135.3	c	0.8	−143.2	b	2.0	63.5	b	3.8	71.7	d	0.5
Koshihikari (premium *japonica* rice)	337.5	f	2.8	73.2	c	0.1	264.3	e	2.8	152.6	c	0.6	−184.9	b	2.2	67.0	c	0.0	79.4	d	0.6
Jasmin rice (low-amylose *indica* rice)	443.1	g	14.8	102.8	e	0.2	340.3	f	15.0	189.6	d	2.1	−253.5	a	12.7	72.0	c	0.5	86.7	d	2.4
Calrose (*japonica* rice)	333.9	f	1.8	82.1	d	0.2	251.8	e	1.5	169.0	d	0.7	−164.9	b	1.1	69.8	c	2.0	86.9	d	0.5
Carnaroli (tropical *japonica* rice)	344.3	f	11.1	94.5	e	0.7	249.9	e	10.4	243.8	e	4.0	−100.5	c	7.1	64.5	b	1.8	149.4	e	3.4
Hoshiyutaka (*japonica-indica* hybrid rice)	214.5	d	8.3	57.8	c	1.1	156.6	d	7.2	185.5	d	4.1	−29.0	e	4.2	62.9	b	2.6	127.6	e	3.0
Basmati (*indica* rice)	159.5	c	0.4	122.5	e	0.2	37.0	a	0.2	345.6	f	4.7	186.1	g	5.0	78.5	d	2.4	223.2	j	4.8
Goami2 (*Ae* mutant rice)	210.5	d	1.0	134.1	e	0.7	76.4	b	1.7	419.5	g	0.1	209.1	g	1.1	82.1	d	0.8	285.5	k	0.7
Niigata 129gou (*Ae* mutant rice)	186.0	d	0.3	116.7	e	0.2	69.3	b	0.1	333.5	f	3.2	147.6	f	3.5	80.8	d	0.4	216.8	j	3.4
Dodam (*Ae* mutant rice)	114.5	a	1.9	64.6	c	0.8	49.8	a	1.2	235.5	e	0.9	121.0	f	1.1	78.2	d	0.0	170.8	f	0.1
Benika (red glutinous rice)	135.8	a	0.6	25.5	a	0.2	110.4	b	0.8	56.3	b	0.2	−79.6	e	0.3	48.2	a	0.2	30.8	a	0.4
Shihou (purple glutinous rice)	156.1	b	1.0	33.5	b	0.5	122.6	b	1.5	72.6	b	1.1	−83.5	e	0.1	49.4	a	1.2	39.0	b	1.6
Hakuchomochi (glutinous rice)	143.1	b	2.1	16.3	a	0.0	126.8	b	2.1	37.6	a	0.2	−105.5	d	1.9	58.5	b	1.2	21.3	a	0.2
Koganemochi (glutinous rice)	206.8	c	0.2	53.3	c	0.4	153.5	c	0.2	105.8	c	0.7	−101.0	d	0.5	70.8	d	4.9	52.5	c	0.4
Himenomochi (glutinous rice)	107.8	a	2.2	20.5	a	0.1	87.3	a	2.3	45.1	a	0.1	−62.7	f	2.3	64.9	c	1.3	24.6	a	0.0
Kinunohada (glutinous rice)	108.3	a	1.2	21.8	a	0.1	86.5	a	1.4	46.7	a	0.2	−61.6	f	1.5	64.7	c	1.7	24.8	a	0.1
Kitayukimochi (glutinous rice)	124.5	a	1.8	18.5	a	0.5	106.0	b	2.3	40.5	a	1.1	−84.0	e	2.8	55.3	b	3.2	22.0	a	0.5
Yumepirika (low-amylose *japonica* rice)	271.9	d	5.8	40.7	b	0.2	231.2	d	6.0	105.1	c	0.8	−166.8	c	5.1	64.4	c	0.0	64.4	c	0.9
Koshihikari (premium *japonica* rice)	333.6	e	5.7	43.1	b	0.2	290.5	d	5.5	112.5	c	0.4	−221.2	b	5.3	69.6	d	0.4	69.3	d	0.2
Jasmin rice (low-amylose *indica* rice)	434.8	f	0.5	57.4	d	1.8	377.4	e	1.2	128.9	c	2.0	−305.9	a	1.5	71.6	d	2.1	71.5	d	0.2
Calrose (*japonica* rice)	320.2	e	6.0	47.9	c	0.3	272.3	d	5.7	122.3	c	0.7	−197.9	b	5.3	69.0	d	0.0	74.4	d	0.4
Carnaroli (tropical *japonica* rice)	324.5	e	1.9	51.4	c	0.9	273.1	d	2.8	170.1	d	0.7	−154.4	c	2.6	66.1	c	0.0	118.7	f	0.2
Hoshiyutaka (*japonica-indica* hybrid rice)	200.3	c	0.9	33.5	b	0.2	166.8	c	0.8	115.3	c	0.2	−85.0	e	0.7	64.1	c	0.0	81.8	e	0.1
Basmati (*indica* rice)	159.3	b	0.1	81.0	e	1.0	78.2	a	1.0	228.4	e	1.5	69.1	i	1.4	70.6	d	8.8	147.3	g	0.5
Goami2 (*Ae* mutant rice)	209.1	c	2.7	60.6	d	0.9	148.5	c	1.8	205.2	e	2.5	−3.9	h	0.2	82.6	e	0.6	144.5	g	1.6
Niigata 129gou (*Ae* mutant rice)	184.5	c	1.1	33.8	b	1.1	150.8	c	0.1	115.5	c	2.2	−69.0	f	1.1	79.0	e	0.3	81.8	e	1.1
Dodam (*Ae* mutant rice)	116.5	a	3.4	18.2	a	0.2	98.3	a	3.2	75.0	b	0.5	−41.4	g	3.9	76.6	e	0.4	56.8	c	0.7

Within each measure (Max. vis, Mini. vis, etc.) in the same column, different letters (a, b, c, etc.) denote statistically significant differences.

**Table 3:** Physical properties of cooked rice of various kinds of cultivars.

Sample	Surface Layer Hardness (H1)		SD	Overall Hardness (H2)		SD	Surface Layer Stickiness (S1)		SD	Overall Stickiness (S2)		SD	Surface Layer Adhered (L3)		SD	Surface Layer Balance Degree H1		SD	Overall Balance Degree H2		SD	Surface Layer Balance Degree A1		SD	Overall Balance Degree A2		SD
	×10^5^ [N/cm^2^]			×10^5^ [N/cm^2^]			×10^5^ [N/cm^2^]			×10^5^ [N/cm^2^]			[mm]			(S1/H1)			(S2/H2)			(A3/A1)			(A6/A4)		
Benika (red glutinous rice)	0.03	a	0.01	1.26	a	0.14	−0.004	b	0.002	−0.299	a	0.048	0.0030	c	0.0005	0.13	c	0.06	0.24	b	0.04	0.49	c	0.27	0.16	c	0.06
Shihou (purple glutinous rice)	0.04	a	0.01	1.32	a	0.13	−0.006	b	0.003	−0.321	a	0.032	0.0028	c	0.0006	0.17	c	0.07	0.24	b	0.02	0.58	c	0.31	0.19	c	0.06
Hakuchomochi (glutinous rice)	0.04	a	0.01	1.10	a	0.25	−0.005	b	0.002	−0.265	a	0.085	0.0032	c	0.0000	0.14	c	0.06	0.23	b	0.04	0.54	c	0.27	0.19	c	0.06
Koganemochi (glutinous rice)	0.03	a	0.01	1.17	a	0.12	−0.006	b	0.003	−0.277	a	0.039	0.0031	c	0.0001	0.18	c	0.08	0.24	b	0.02	0.74	c	0.39	0.21	c	0.06
Himenomochi (glutinous rice)	0.04	a	0.01	1.12	a	0.15	−0.005	b	0.003	−0.286	a	0.055	0.0031	c	0.0001	0.14	c	0.06	0.25	b	0.03	0.55	c	0.32	0.18	c	0.07
Kinunohada (glutinous rice)	0.04	a	0.01	1.15	a	0.12	−0.005	b	0.002	−0.301	a	0.050	0.0031	c	0.0004	0.12	c	0.04	0.26	b	0.03	0.45	c	0.25	0.17	c	0.04
Kitayukimochi (glutinous rice)	0.05	a	0.01	1.20	a	0.11	−0.006	b	0.002	−0.332	a	0.037	0.0032	c	0.0000	0.13	c	0.04	0.28	b	0.02	0.39	c	0.17	0.16	c	0.05
Yumepirika (low-amylose japonica rice)	0.06	b	0.02	1.58	b	0.19	−0.006	b	0.004	−0.369	a	0.072	0.0024	b	0.0010	0.10	c	0.05	0.23	b	0.04	0.16	b	0.11	0.11	b	0.04
Koshihikari (premium *japonica* rice)	0.07	b	0.02	1.67	b	0.16	−0.010	a	0.006	−0.337	a	0.066	0.0031	c	0.0002	0.15	c	0.07	0.20	b	0.04	0.26	b	0.12	0.10	b	0.04
Jasmin rice (low-amylose *indica* rice)	0.07	b	0.02	1.65	b	0.19	−0.006	b	0.003	−0.351	a	0.056	0.0025	b	0.0009	0.08	b	0.05	0.22	b	0.04	0.15	b	0.09	0.08	b	0.02
Calrose (*japonica* rice)	0.09	c	0.03	1.68	b	0.23	−0.007	b	0.004	−0.362	a	0.073	0.0026	c	0.0009	0.07	b	0.04	0.22	b	0.05	0.13	b	0.09	0.08	b	0.04
Carnaroli (tropical *japonica* rice)	0.10	c	0.04	2.89	e	0.52	−0.004	b	0.004	−0.173	b	0.076	0.0024	b	0.0010	0.04	a	0.02	0.06	a	0.03	0.07	a	0.04	0.03	a	0.01
Hoshiyutaka (*japonica-indica* hybrid rice)	0.09	c	0.02	2.10	c	0.26	−0.003	c	0.002	−0.139	b	0.073	0.0020	b	0.0012	0.03	a	0.02	0.07	a	0.04	0.06	a	0.03	0.03	a	0.01
Basmati (*indica* rice)	0.12	d	0.02	2.26	d	0.25	−0.001	c	0.001	−0.091	c	0.070	0.0013	a	0.0011	0.01	a	0.01	0.04	a	0.03	0.02	a	0.03	0.01	a	0.01
Goami2 (*Ae* mutant rice)	0.18	e	0.05	2.35	d	0.30	−0.001	d	0.000	−0.005	d	0.007	0.0009	a	0.0010	0.00	a	0.00	0.00	a	0.00	0.01	a	0.01	0.00	a	0.01
Niigata 129gou (*Ae* mutant rice)	0.23	e	0.06	2.38	d	0.35	−0.001	d	0.000	−0.024	d	0.015	0.0012	a	0.0012	0.01	a	0.00	0.01	a	0.01	0.01	a	0.01	0.01	a	0.01
Dodam (*Ae* mutant rice)	0.21	e	0.08	2.38	d	0.41	−0.001	d	0.000	−0.014	d	0.048	0.0016	a	0.0012	0.01	a	0.00	0.01	a	0.03	0.01	a	0.01	0.01	a	0.01

Within each measure (H1, H2, etc.) in the same column, different letters (a, b, c, etc.) denote statistically significant differences.

**Table 4:** Correlation between the pasting properties of the three programs with the results of the physical parameters of cooked rice, iodine analysis, RS content, and prolamin ratio of 17 rice cultivars.

	H1(R)		H2(R)		H2(R.D)		S2(R.D)		Aλ_max_ (starch)		Aλ_max_ (cooked)		RS		Ploramin	
Consistency (93 °C)	0.53	*	0.75	**	0.47		−0.57	*	0.50	*	0.50	*	0.24		0.74	**
Consistency (120 °C)	0.92	**	0.90	**	0.72	**	−0.92	**	0.85	**	0.88	**	0.80	**	0.51	*
Consistency (140 °C)	0.67	**	0.82	**	0.56	*	−0.72	**	0.58	*	0.61	**	0.43		0.62	**

Correlation is significant at 5% (*) or (**) by the method of *t*-test.

## Data Availability

The datasets generated for this study are available on request to the corresponding author.

## Supplementary Materials

The following are available online at https://www.mdpi.com/article/10.3390/foods10050987/s1, Figure S1: SDS-PAGE analysis of proteins extracted from 17 rice samples; Table S1: Physical properties of cooked rice after 24 h at 6 °C.

## Abbreviations

AAC: apparent amylose content; λ_max_: peak wavelength on iodine staining; Aλ_max_: absorbance at λ_max_; Fb_3_, proportions of long chains in amylopectin (DP > 37%); CD, chain length distribution; SD, standard deviation; RS, resistant starch; SLC, super-long chains; RVA, rapid visco analyzer; SB, setback; BD, breakdown; Max. vis., maximum viscosity; Mini. vis., minimum viscosity; Pt, pasting temperature; Cons, consistency; Fin. vis, final viscosity; H1, the hardness of the surface layer; H2, hardness of the overall layer; S1, the stickiness of the surface layer; S2, the stickiness of the overall layer; L3, the adhesion of the surface layer; Balance H1, the ratio of stickiness to hardness of the surface layer; Balance H2, the ratio of stickiness to hardness of the overall layer; Balance A1, the ratio of adhesive work to hardness work of the surface layer; Balance A2, the ratio of adhesive work to hardness work of the overall layer; H1(R), retrogradation of surface layer; H2(R), retrogradation of overall hardness; H1(R.D), retrogradation degree of surface layer hardness; S2(R.D), retrogradation degree of overall stickiness; Program 1, program of maintenance for 7 min at 93 °C; Program 2, program of maintenance for 2 min at 120 °C; Program 3, program of maintenance for 3.3 min at 140 °C.; RVA, rapid visco analyzer; SDS-PAGE, sodium dodecyl sulfate polyacrylamide gel electrophoresis; DP, degree of polymerization; AOAC, Association of Offficial Analytical Collaboration International.
