# Supplementary material for: Evaluation of Hardness and Retrogradation of Cooked Rice Based on Its Pasting Properties Using a Novel RVA Testing
Source: Foods. 2021 Apr 30;10(5):987. doi: 10.3390/foods10050987 (PMC8147165; doi:10.3390/foods10050987)
Supplement: Supplementary file 1 [file foods-10-00987-s001.zip › foods-1189722-supplementary.pdf]

Supplemental Table 1. Physical properties of cooked rice after 24h at 6°C.

Sample	Surface layer Hardness (H 1) $\times10^5$[N/cm2]		SD	Overall Hardness (H 2) $\times10^5$[N/cm2]		SD	Surface layer Stickiness (S 1) $\times10^5$[N/cm2]		SD	Overall Stickiness (S 2) $\times10^5$[N/cm2]		SD	Surface layer Adhered (L 3) [mm]		SD	Surface layer Balance degree H1 (S1/H1)		SD	Overall Balance degree H2 (S2/H2)		SD	Surface layer Balance degree A1 (A3/A1)		SD	Overall Balance degree A2 (A6/A4)		SD
Benika (red glutinous rice)	0.03	a	0.01	1.04	a	0.12	-0.003	b	0.002	-0.266	c	0.052	0.0029	e	0.0005	0.12	c	0.05	0.26	c	0.03	0.39	d	0.10	0.15	c	0.04
Shihou (purple glutinous rice)	0.04	a	0.01	1.21	a	0.15	-0.004	b	0.003	-0.295	b	0.035	0.0029	e	0.0005	0.11	c	0.06	0.25	c	0.03	0.37	d	0.24	0.14	c	0.05
Hakuchomochi (glutinous rice)	0.04	a	0.01	1.20	a	0.08	-0.006	a	0.001	-0.313	b	0.036	0.0032	e	0.0000	0.17	d	0.05	0.26	c	0.02	0.69	e	0.29	0.21	c	0.04
Koganemochi (glutinous rice)	0.04	a	0.01	1.22	a	0.13	-0.007	a	0.002	-0.312	b	0.047	0.0031	e	0.0001	0.18	d	0.06	0.25	c	0.02	0.54	d	0.28	0.18	c	0.06
Himenomochi (glutinous rice)	0.04	a	0.01	1.17	a	0.11	-0.007	a	0.002	-0.315	b	0.038	0.0032	e	0.0001	0.18	d	0.05	0.27	c	0.03	0.77	e	0.36	0.25	c	0.09
Kinunohada (glutinous rice)	0.04	a	0.01	1.18	a	0.15	-0.006	a	0.002	-0.317	b	0.040	0.0032	e	0.0000	0.14	c	0.04	0.27	c	0.02	0.52	d	0.28	0.18	c	0.06
Kitayukimochi (glutinous rice)	0.04	a	0.01	1.18	a	0.10	-0.006	a	0.002	-0.318	b	0.037	0.0031	e	0.0002	0.15	c	0.04	0.27	c	0.02	0.44	d	0.15	0.18	c	0.04
Yumepirika (low-amylose japonica rice)	0.08	b	0.02	1.72	b	0.24	-0.006	a	0.004	-0.426	a	0.045	0.0025	d	0.0009	0.08	b	0.05	0.25	c	0.04	0.13	c	0.08	0.08	b	0.03
Koshihikari (premium japonica rice)	0.10	b	0.03	1.70	b	0.21	-0.003	b	0.002	-0.238	c	0.100	0.0023	c	0.0009	0.04	b	0.03	0.15	b	0.07	0.06	b	0.03	0.05	b	0.02
Jasmin rice (low-amylose indica rice)	0.08	b	0.02	1.62	b	0.19	-0.004	b	0.003	-0.291	b	0.085	0.0028	e	0.0007	0.05	b	0.04	0.19	b	0.07	0.10	c	0.06	0.06	b	0.02
Calrose (japonica rice)	0.15	c	0.06	2.03	c	0.21	-0.006	a	0.005	-0.336	b	0.085	0.0021	c	0.0011	0.05	b	0.04	0.17	b	0.05	0.08	c	0.06	0.05	b	0.03
Carnaroli (tropical japonica rice)	0.14	c	0.06	3.44	f	0.66	-0.001	c	0.001	-0.063	e	0.075	0.0010	a	0.0010	0.01	a	0.01	0.02	a	0.03	0.01	a	0.02	0.01	a	0.01
Hoshiyutaka (japonica-indica hybrid rice)	0.12	c	0.03	2.10	c	0.22	-0.001	d	0.000	-0.111	d	0.066	0.0016	b	0.0012	0.01	a	0.00	0.05	a	0.03	0.02	a	0.02	0.02	a	0.01
Basmati (indica rice)	0.23	d	0.10	2.57	d	0.38	-0.001	d	0.000	-0.010	f	0.014	0.0008	a	0.0010	0.00	a	0.00	0.00	a	0.01	0.01	a	0.01	0.00	a	0.01
Goami2 (Ae mutant rice)	0.29	e	0.09	3.09	e	0.36	-0.001	d	0.000	0.000	f	0.000	0.0005	a	0.0009	0.00	a	0.00	0.00	a	0.00	0.00	a	0.01	0.00	a	0.01
Niigata 129gou (Ae mutant rice)	0.33	e	0.10	2.92	e	0.37	-0.001	d	0.000	-0.001	f	0.002	0.0009	a	0.0012	0.00	a	0.00	0.00	a	0.00	0.00	a	0.01	0.00	a	0.01
Dodam (Ae mutant rice)	0.32	e	0.08	2.89	e	0.30	-0.001	d	0.000	0.000	f	0.000	0.0014	b	0.0013	0.00	a	0.00	0.00	a	0.00	0.01	a	0.01	0.01	a	0.01

Within each measure (H1, H2, etc.) in the same column, different letters (a, b, c, etc.) denote statistically significant differences.

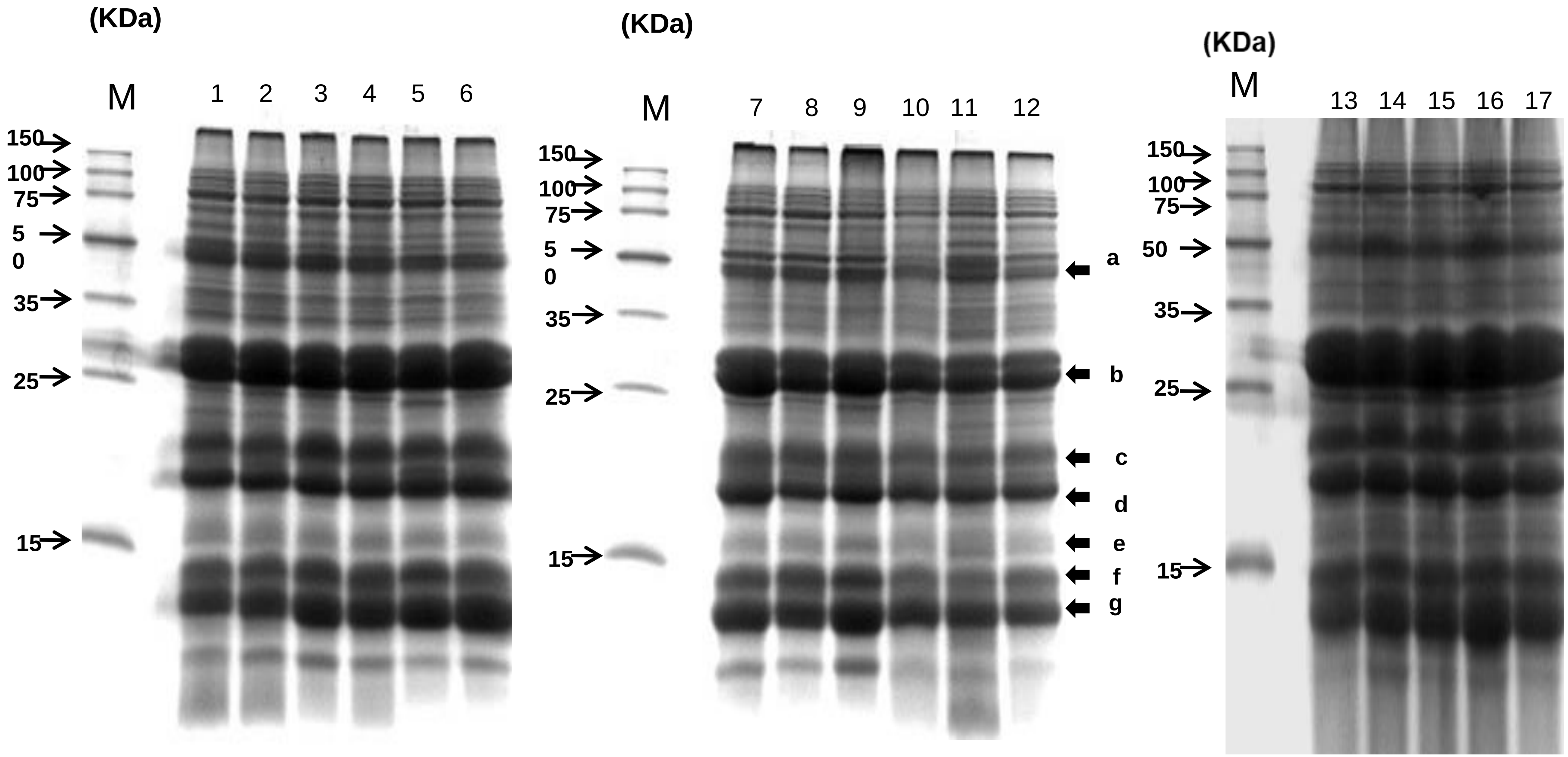

a; Gluterin precursor, b; Glutelin α-subunit, c; α-Globulin, d; Glutelin β-subunit, e; f; g; Prolamin

1, Benika; 2, Shihou; 3, Yumepirika; 4, Koshihikari; 5, Jasmin rice; 6, Calrose;
7, Carnaroli; 8, Hoshiyutaka; 9, Basmati; 10, Goami2; 11, Niigata129go; 12, Dodam;
13,Koganemochi; 14, Hakuchomochi; 15, Himenomochi; 16, Kitayukimochi; 17, Kinunohada

Supplemental Fig.1 SDS-PAGE analysis of proteins extracted from 17 rice samples.
